# Clinical manifestations in infants and children with *Mycoplasma pneumoniae* infection

**DOI:** 10.1371/journal.pone.0195288

**Published:** 2018-04-26

**Authors:** Mia Johanna Søndergaard, Martin Barfred Friis, Dennis Schrøder Hansen, Inger Merete Jørgensen

**Affiliations:** 1 Department of Paediatrics, Nordsjællands Hospital, Hillerød, Denmark; 2 Department of Clinical Microbiology, Herlev Hospital, Herlev, Denmark; Kliniken der Stadt Köln gGmbH, GERMANY

## Abstract

**Background:**

*Mycoplasma pneumoniae* is a common cause of community-acquired pneumonia in older children. Pulmonary and extra-pulmonary symptoms associated with *M*. *pneumoniae* infection are reported. *M*. *pneumoniae* is mainly epidemic in Denmark with the recurrence every 4-7^th^ year.

**Aims:**

Retrospectively, to describe the epidemiology and clinical features, in infants and children, during the *M*. *pneumoniae* epidemic in 2010 and 2011.

**Methods:**

All children under the age of 16 that were tested for *M*. *pneumoniae* during the period 01.02.2010–31.01.2012 were included. Medical charts, as well as radiological findings, were reviewed for all children with *M*. *pneumoniae*. A post-hoc analysis of viral co-infections was done on part of the cohort.

**Results:**

134 of 746 children were tested positive for *M*. *pneumoniae* by PCR or serology. Positive tests were found in 65% of children seven years and older, in 30% of 2-6-year-olds and 4% of infants (less than two years of age). Viral co-infection was found in 27% of the tested samples. The clinical presentation was a cough, asthma-like symptoms and low-grade fever. Extra-pulmonary symptoms were common and presented as nausea/vomiting by 33% of the children and skin manifestations by 25%. 84% of the children had a chest x-ray taken, and there were positive radiological findings in 94% of these.

**Conclusion:**

*M*. *pneumoniae* also affected infants and young children and symptoms were similar to infections with respiratory viruses, but severe LRTI were also seen. During an up-coming epidemic, assessment of extra-pulmonary manifestations can be helpful when diagnosing *M*. *pneumoniae* infections.

## Introduction

*Mycoplasma pneumoniae* (*M*. *pneumoniae*) is an important cause of respiratory tract infections, especially in children and younger adults and are estimated to be accountable for up to 30–40% of community-acquired pneumonia (CAP) [1–4]. Pulmonary manifestations are typically tracheobronchitis and pneumonia accompanied by a cough but also wheezing. Young children are considered not to be as susceptible to *M*. *pneumoniae* as school-aged children [5,6]. In Denmark, *M*. *pneumoniae* is mainly epidemic with a recurrence rate every 4-7^th^ year [7].

Denmark experienced an *M*. *pneumoniae* epidemic, peaking in the autumn of 2010 and again in the autumn of 2011. Statens Serum Institut (SSI), responsible for the national surveillance system in Denmark, has described it as two waves of the same epidemic [7].

The realisation that also pre-school children and even infants can be susceptible and have clinical symptoms from *M*. *pneumoniae* infection has evolved during recent years and has been reported in studies from Europe and Australia [8–14]. From a clinical view, it is essential to establish if *M*. *pneumoniae* plays a significant differential role in lower respiratory tract infections and asthma-like exacerbations in young children and infants. In recent years the issue of co-infections and non-symptomatic carriage of *M*. *pneumoniae* and hence over-diagnosing of *M*. *pneumoniae* by PCR has been debated [15].

The purpose of this study was to describe and characterise the *M*. *pneumoniae* epidemic in a hospital setting, and to evaluate possible age-dependent clinical features in infants, young children and older children hospitalised and diagnosed with an *M*. *pneumoniae* infection. The study was set up since we experienced a shift in the clinical picture of our *M*. *pneumoniae* patients during the epidemic. The literature on this area is extensive with varying opinions, as mentioned, on the pathogenicity of the bacteria and the indication for treatment as well as on the significance of infections in pre-school children. Our report is another brick in understanding this pathogen.

## Materials and methods

We designed a retrospective analysis of all children younger than 16 years of age admitted to the Department of Paediatrics, Nordsjællands Hospital, Hillerød, Denmark, that were investigated for *M*. *pneumoniae* either by polymerase chain reaction (PCR) or by serology (*M*. *pneumoniae* antibody test and cold-agglutinin test). The study period was 01.02.2010 to 31.01.2012. We collected PCR results and blood sample results from both our local microbiology laboratory (PCR) and from SSI (PCR and serology). Data were matched on social security number to avoid double sampling. PCR was performed on oropharyngeal-swabs that were stored and transported cooled to the laboratory. Diagnosis of *M*. *pneumoniae* was based on a commercially available real-time-PCR (RT-PCR) kit (Minerva Biolabs Venor^R^ Mp *Mycoplasma pneumoniae* -Diagnostic Kit for qPCR type I) targeting the P1 cytoadhesion gene. The PCR was a multiplex analysis also targeting *Chlamydophila pneumoniae/psittaci*. The assay was performed in accordance with the manufactures description. In short 10μl sample was added to 14.4μl mastermix containing 14μl buffer, 0.2μl Taq-polymerase and 0.2μl Uracil-N-Glycosylase. RT-PCR was performed under the following conditions on a Stratagene Mx 3005P RT-PCR machine: 10 min at 95°C followed by 45 cycles of 30 sec at 95°C, 30 sec at 55°C and 30 sec at 60°C. *M*. *pneumoniae* was detected in the FAM filter.

The serological tests were performed by an external commercial provider (SSI) and were based on *M*. *pneumoniae* specific IgM antibodies (MPT) together with cold-agglutinins (KAT). A titre of both MPT and KAT above or equal to 64 was considered as a positive test. Since the KAT titres fall more rapidly after a passed infection, it was used to strengthen the likelihood of an ongoing infection. It is estimated that 95% of persons under the age of 20 have positive KAT titres in response to an *M*. *pneumoniae* infection (SSI test-information).

Children who presented with two or more positive PCR-tests within three months were considered as the same infective episode and only included once.

The children were referred by general practitioners or the hospital's emergency department. The doctor on call ordered the *M*. *pneumoniae* sampling according to the clinical evaluation.

Demographic and clinical data were collected for the 134 children, who tested positive for *M*. *pneumoniae*, by medical chart review. The charts of 612 children with negative tests have not been audited. Medical history was systematically reviewed using computerised medical records, radiological reports and laboratory reports. Characteristics analysed included demographics (age, gender, medical history prior to admission), clinical presentation (pulmonary and extra-pulmonary symptoms, clinical examination; respiratory rate, auscultation, temperature, oxygen saturation), radiological findings, biochemistry (C-reactive protein (CRP), leucocyte count at admission) co-infections, complications and medical treatment. Children were divided in age-groups for data analysis, less than two years old (referred to in the text as infants), 2-6-year-olds and 7-15-year-olds (school-aged children).

To further meet the question of viral co-infections, a post-hoc analysis of the original, frozen, oropharyngeal swab specimen, from 49 of the *M*. *pneumoniae* positive cohort, were tested by PCR using a commercially available kit (Biomerieux Respiratory MWS R-Gene range) for a wide range of respiratory viruses (including; respiratory syncytial virus (RSV), influenza A and B, human metapneumovirus, rhinovirus, parainfluenza virus, coronavirus, bocavirus and adenovirus). In short, PCR was performed in four dual-plexes; a 10μl sample was added to 15μl mastermix containing a buffer, Reverse transcriptase and primer/probe-mix. RT-PCR was performed, under the following conditions, on a Stratagene Mx 3005P RT-PCR machine: 5 min at 50°C, 15 min at 95°C followed by 45 cycles of 10 sec at 95°C, 40 sec at 60°C and 25 sec at 72°C. Al targets were detected in the either the FAM or HEX filter.

Samples positive after more than 35 Ct-cycles were re-evaluated to confirm a true positive result.

The Ethical Committee of Region Hovedstaden (protocolnumber: H-2-2012-132) as well as the Danish Data Protection Agency approved the study. This approval included acceptance of reviewing the medical records without informed consent of the patients.

Statistical analyses were performed in STATA 10.0, software packages SPSS version 22 and Microsoft Excell. Chi^2^-test, Fishers Exact test or Z-score test was used for categorical data analysis and two-sample T-test for numerical data. A p-value of < 0,05 was considered significant.

## Results

### Epidemiological findings

Out of 885 patients tested for *M*. *pneumoniae* in the study period, 139 were excluded due to age (older than 15 years of age), double sampling or repeated samples within three months. A total of 746 children with respiratory tract infection (RTI) were enrolled in the study and 134 had a positive test for *M*. *pneumoniae*. Out of 740 *M*. *pneumoniae* PCR analyses included, 132 were positive and out of 20 serological tests, 3 were positive. Fourteen children were tested with both real-time PCR and serology.

**Fig 1:**
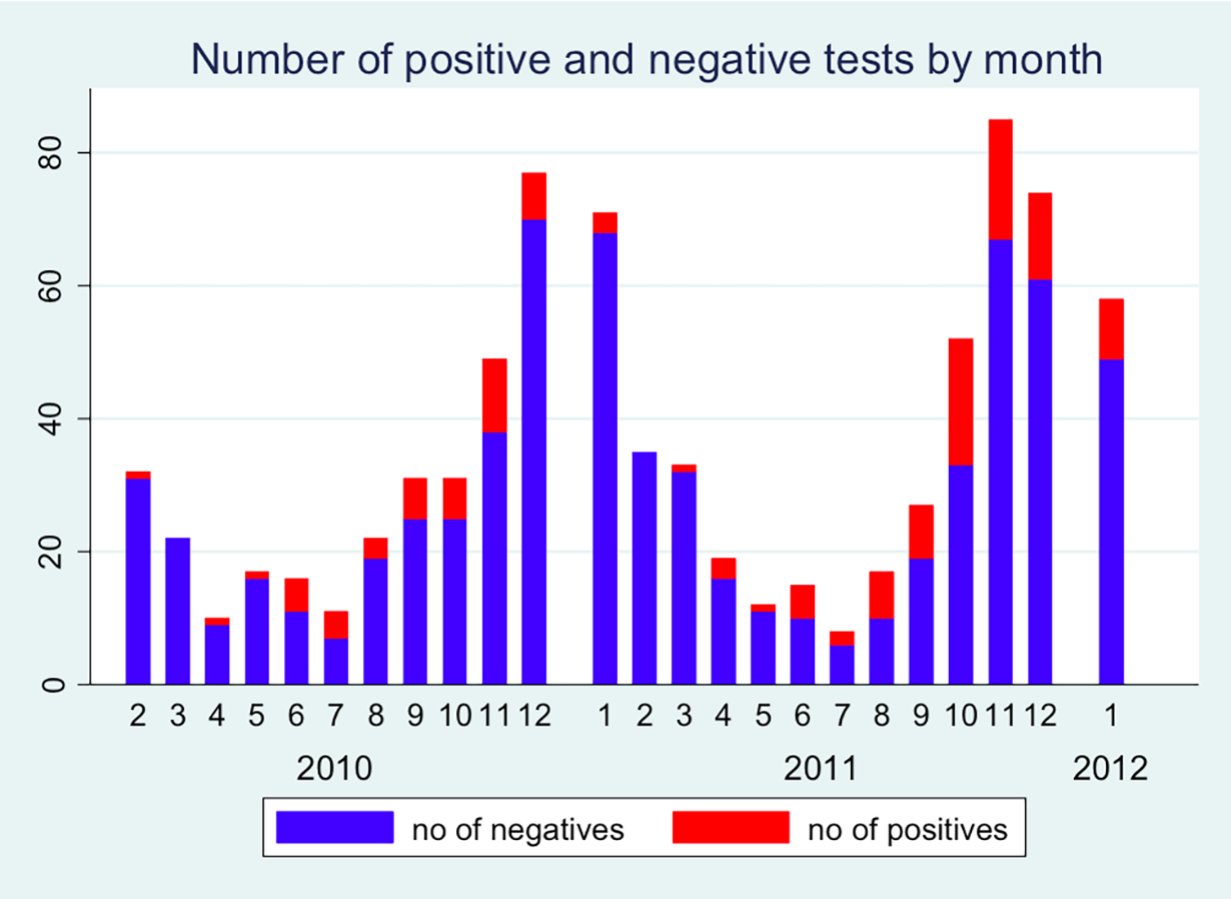
Number of positive and negative *M*. *pneumoniae* tests by year and month.

Fig 1 shows the number of children tested for *M*. *pneumoniae* and the numbers of positive cases by month. The epidemic peaked in October 2010 and again in October to December 2011. The number of *M*. *pneumoniae* positive cases was 50 (of 386) in the first epidemic season and 84 (of 360) in the second period of the epidemic, with a significantly higher rate (0,13 vs 0,23) of positive cases in the later (p = 0,0002, chi^2^-test). Overall 18% of the children tested for M. pneumonia had a positive sample.

The highest rate of *M*. *pneumoniae* positive samples was in school-aged children (65%) but notably also pre-school children (30%), and even six children under the age of two (4%) had *M*. *pneumoniae* positive tests.

### Clinical presentation, radiological findings and laboratory test

Patient characteristics stratified by age group are presented in Table 1.

**Table 1:** Symptoms, extrapulmonary symptoms, objective signs, biochemistry and radiological findings in children with Mycoplasma pneumoniae.

	<2 y	2–6 y	7–15 y	Total		<2 y	2–6 y	7–15 y	Total
					**Objective signs:**				
Number of children	6	40	88	134	Tachypnea, (%)^W^	0	52	57	52
Male (%)	67	60	53	56	Temperature °C (median [IQR])	38.5 [37.4:39.4]	37.8 [37.3:38.5]	37.9 [37.5:38.8]	37.9 [37.4:38.8]
Symptomatic days before admission (mean)*	4	12	10	10	Auscultation—wheezing (%)	33	24	23	24
Days of admission (mean)**	2	1,8	2,3	2,2	Auscultation—crackles/decreased breath sounds (%)	100	47	57	56
**Symptoms:**					**Laboratory Tests:**^***Y***^				
Cough (%)	100	100	99	99	CRP, mg/L (median [IQR])	33 [too few]	28.5 [17.5:40.3]	25 [16.5:32.5]	28 [16.3:39.3]
Wheezing (%)	50	17	16	17	Leukocyte count (median [IQR])	17.0 [too few]	8.1 [5.8:11.9]	10.3 [8.3:12.7]	9.3 [6.2:12.2]
Rhinorrhoea (%)	40	19	11	15	Neutrophil count (median [IQR])	9.6 [too few]	5.3 [3.9:9.3]	6.6 [5.1:8.3]	5.8 [4.0:8.9]
Sore throat (%)	0	18	26	22	Lymphocyte count (median [IQR])	5.2 [too few]	1.5 [1.1:1.8]	2.2 [1.7:2.9]	1.7 [1.3:2.5]
Croup symptoms (%)	0	0	5	3	*** ***				
**Extrapulmonary symptoms:**					**Radiological findings:**^**Z**^				
Fever ≥ 38°C (%)	83	94	90	91	Hilar adenopathy (exclusively) (%)	33	13	5	8
Skin manifestation (all)^X^ (%)	33	18	24	23	Lobar infiltration (%)	33	81	82	80
Urticarial rash (%)	17	0	13	9	Atelectasis (%)	0	16	19	18
Steven Johnson syndrome (no)	0	0	2	2	Pleural effusions (%)	0	9	9	9
Nausea and/or vomiting (%)	33	32	30	31	Empyema (no)	0	0	1	1

*Children under the age of two are seen significantly earlier than older children (p = 0.01, two-sample t-test).

**Tendency of longer hospitalisations among older children. ^W^- Tachypnea was defined as a respiratory rate over the 99.th percentile for the child's age [16]. ^**X**^—including any type of rash, urticaria and Steven Johnson syndrome. ^**Y**^—81 children had a blood sample drawn. ^**Z**^—112 children had a chest x-ray taken; only 7 were described as normal

Children under two years of age were admitted to the hospital significantly earlier after the onset of symptoms than older children, (p = 0.01, two-sample t-test). 61% of the children were discharged from hospital on the day of admission.

The most common respiratory manifestation was a cough (100%) together with an age-depending degree of wheezing (Table 1). At admission to the hospital, 92% of infants and children were diagnosed with an episode of troublesome lung symptoms lasting at least three days. Significantly more young children (less than three years of age) had objective wheezing and cough (asthma-like symptoms) than older children (p = 0.01, Z-score test). Previous and clinically significant chronic disease was diagnosed in 10% of the children. Of these 4/13 (31%) had previously diagnosed and current asthma, and had a severe exacerbation during the *M*. *pneumoniae* infection.

The majority of the patients (84%) had a chest x-ray taken, and 96% of these had positive radiological findings (Table 1). Among infants and young children, exclusive hilar adenopathy was more frequent, while older children usually had significant peripheral infiltration on the chest X-ray (Table 1). Children with atelectasis had a significantly longer duration of hospital stay; more than three days (35% versus 25%; p = 0.05). The rate of pulmonary complications was the same for children with CRP over 50 mg/l (POCT(Point of Care Testing), part of an adult definition of significant pneumonia) as below 50 mg/l (18% versus 19%, p > 0.05). No pulmonary complications were reported in the under two age-group. The number of severe manifestations of pneumonia was equal in the age-groups; 2–6 years and 7–15 years (Tabel 1). Overall 20% of the children had an increased CRP level of more than 50 mg/l, and they were all older than three years of age.

A total of 120 children received antibiotic treatment. The majority were treated with clarithromycin according to the local Guideline. Sixty-four patients, or 46%, were treated upon suspicion. Out of these, 53% had received other antibiotics (beta-lactam) prior to *M*. *pneumoniae* testing. Fifty-six patients (42%) were started on treatment upon receiving the positive test result. Only six children were treated with macrolide antibiotics twice due to suspicion of recurrence or treatment failure. One sample was tested for macrolide resistance and found negative.

The most common extra-pulmonary manifestation was nausea, with or without vomiting, reported by a third of all children. 23% of all children had some type of rash, and 9% had hives. In infants, there were skin manifestations in 33% of the cases. Two children developed Steven Johnson syndrome (SJS) with mucosal symptoms arising prior to or at the same time as the antibiotic treatment was started.

A total of 37 children had simultaneously been tested with sputum-culture for other bacterial pathogens. In 41% of these, a co-infection was diagnosed. The most common bacteria were *Moraxella catarrhalis*, *Haemophilus influenzae* and *S*. *pneumonia*. Due to methodological setup, all children were tested for *Chlamydophila pneumoniae* none were found positive.

Only two children were tested for viral infections during the clinical setup. The post-hoc analyses of 49 oropharyngeal-swabs showed that 27% of these had a single or mixed viral co-infection (RSV (1 child), influenza A (2), human metapneumovirus (1), rhinovirus (2), coronavirus (3), bocavirus (2) and adenovirus (5)). Four children were PCR positive for two viruses as well as for *M*. *pneumoniae*. Table 2 shows, in a similar matter as Table 1, the clinical characteristics of children with, without and of unknown viral infection. The data suggest that significantly more children with mixed infection of *M*. *pneumoniae* and a respiratory virus had rhinorrhoea (p = 0.02), and were wheezing (tendency, p = 0.07), compared to those who were only positive for *M*. *pneumoniae*. We could not identify other differences between the two groups, including no radiological discrepancies.

**Table 2:** Symptoms, extrapulmonary symptoms, objective signs and radiological findings in children with Mycoplasma pneumoniae with or without a viral co-infection.

	MP positive and virus positive		MP positive and virus negative		MP positive and virus unknown	
	< 2 y	2–15 y	< 2 y	2–15 y	< 2 y	2–15 y
Number of children	3	10	0	36	3	82
Male (%)	33	50	-	64	100	51
Symptomatic days before admission (mean)	4	16	-	9.1	2.7	9.8
Days of admission (mean)	1.8	2.5	-	2.4	1.7	2.2
						
***Symptoms*:**						
Cough (%)	100	90	-	100	100	100
Wheezing (%)	50	50*	-	19*	50	10
Rhinorrhea (%)	0	44**	-	7**	67	14
Sore throat (%)	NA	22	-	27	0	20
Croup symptoms (%)	0	0	-	0	0	7
						
***Extrapulmonary symptoms*:**						
Fever ≥ 38°C (%)	100	70	-	97	67	93
Skin manifestation (all)^X^ (%)	67	0	-	14	0	29
Urticarial rash (%)	33	0	-	3	0	12
Steven Johnson syndrome (no)	0	0	-	1	0	1
Nausea and/or vomiting (%)	0	11	-	39	67	31
						
***Objective signs*:**						
Tachypnea^W^ (%)	0	44	-	53	0	57
Temperature °C (mean)	38.4	38.1	-	38.2	38.2	37.9
Auscultation—wheezing (%)	33	20	-	24	0	27
Auscultation—crackles/decreased breath sounds (%)	100	60	-	52	67	44
						
***Radiological findings*:**						
Hilar adenopathy (exclusively) (%)	50	13	-	7	NA	7
Lobar infiltration (%)	50	63	-	86	NA	85
Atelectasis (%)	0	13	-	25	NA	19
Pleural effusions (%)	0	0	-	11	NA	7
Empyema (no)	0	0	-	0	NA	1
						

Mycoplasma pneumoniae

* Children with a viral co-infection tendency of higher rates of wheezing (p = 0.07).

** Children with a viral co-infection had significantly higher rates of runny noses, p: 0.02 ^W^- Tachypnea was defined as a respiratory rate over the 99.th percentile for the child's age [16]. ^**X**^—including any type of rash, urticarial rash and Steven Johnson syndrome.

## Discussion

We present data from a large cohort of children with *M*. *pneumoniae* infection. All children enrolled were referred from the primary healthcare system to hospitalisation due to character and severity of symptoms. The majority of *M*. *pneumoniae* PCR test-positive children had LRTI, which we confirmed by a high rate of radiological findings (94%).

We found a higher rate of positive samples in the later wave of the epidemic in 2010 and 2011. School-aged children were more often *M*. *pneumoniae* positive (65%) than younger children, but even amongst the 2 to 6-year-old children 30% were *M*. *pneumoniae* positive substantiating our initial suspicion that *M*. *pneumoniae* also affects small children. Even a small number of infants; 6 out of 276 were diagnosed.

This study was conducted as a retrospective chart study. The doctor on call decided whom to test for *M*. *pneumoniae*. Children with *M*. *pneumoniae* infection might have been under-diagnosed if they had minor respiratory symptoms especially during the first wave of the epidemic period. Due to commonly held concepts of CAP epidemiology, originally based on a long-term study conducted in primary care from 1963–1975, we expect infants and young children to be under-diagnosed due to selection-bias [6].

Even very young children can become ill from *M*. *pneumoniae* even though it is less common. The differential diagnosis of respiratory viral infections and exacerbation of asthma-like symptoms must be considered. The clinical presentation with a cough, wheezing, low-grade-fever, CRP below 50 mg/L and rhonchi on auscultation in 33% of the youngest children can also be considered as a childhood asthma-like exacerbation, primarily due to viral infection in pre-school children. Indeed, we also had a minor degree of mixed viral co-infections discovered in our post-hoc analysis. It can only be speculated in what pathogen was the primary cause of disease in these cases. Due to our post-hoc findings, we would advise that small children with wheezing and rhinorrhoea should be tested for both *M*. *pneumoniae* and respiratory viral infections simultaneously. During the Norwegian *M*. *pneumoniae* epidemic, Inchly et al. described a similar relative number of viral co-infections [14].

In a Dutch childhood study of carriage of *M*. *pneumoniae* in the upper respiratory tract (URT), season and year of enrolment affected the prevalence of asymptomatic carries ranging from 3% to 52% [17]. In our study, some of the children discharged from the ward on the same day as admitted to the hospital could have been carriers of *M*. *pneumoniae*. However, several of these children were treated with first-line antibiotics, prior to admission, and referred to our department because of insufficient response to beta-lactam antibiotic management.

Kroppi et al. [18] found that 50% of children with LRTI caused by *M*. *pneumoniae* were co-infected with primarily *S*. *pneumoniae* or *Chlamydiae spp*. Only a small part of this cohort was tested for bacterial co-infection, but we did not regard this as a major problem. Again, it is noteworthy that 59% of the children had been treated with a beta-lactam antibiotic before examination for *M*. *pneumoniae* without improvement of the infection.

In parts of the same epidemic period in Denmark (2010–2011), Stockholm et al. identified an effect of azithromycin (a macrolide antibiotic) on episode duration of asthma-like symptoms in young children. No investigations for *M*. *pneumoniae* were done, and exclusion criteria of respiratory rate over 50/min, temperature over 39°C and CRP over 50 mg/L would not exclude all children with a possible *M*. *pneumoniae* infection [19–21]. Two recently published Norwegian studies described the discrepancy of the incidence of clinical symptomatic *M*. *pneumoniae* infections in preschool children between epidemic and endemic periods [14,21]. Randomised Controlled Trials concerning the efficacy of macrolides on asthma-like symptoms should be conducted in endemic periods or better controlled for *M*. *pneumoniae* infections, especially in young children born after an epidemic period. The anti-inflammatory effect of macrolides still has to be further addressed [22].

10% of this cohort was affected by chronic illness, mainly respiratory severe illness. Severe asthma exacerbations were diagnosed in the current asthmatics.

Older children tended to be seen later after onset of symptoms and were accountable for the longer hospitalisations. This might indicate that older children had more severe infections, or that the delay in admission to the hospital resulted in more severe disease and thereby a prolonged period of rehabilitation. Despite that, we also identified severe pneumonia based on the radiological findings (atelectasis, pleural effusions) in the 2-6-year-olds. If adjusted for population size these preschool children had an increased risk of developing severe pneumonia compared with school children during this epidemic. Inchley et al. showed the same pattern even if their definition of severe pneumonia differed [14]. Treating the infections earlier might reduce severe morbidity and length of hospital stays.

Treatment of *M*. *pneumoniae* infections with macrolide antibiotics is controversial since a Cochrane review, concluded that there is insufficient evidence to draw any specific conclusions about the efficacy of antibiotics in *M*. *pneumoniae* infections in children [17,22]. The efficacy of antibiotic treatment should be discussed in light of a correct diagnostic test [15]. Asymptomatic carriers of *M*. *pneumoniae* have to be differentiated from children suffering from symptomatic infections, LRTI, caused by *M*. *pneumoniae* [23]. Gardiner et al. underline the need for RCT on this topic [17]. In Denmark, SSI still recommends treatment of *M*. *pneumoniae* positive LRTI in children [24]. Macrolide resistance is a growing problem worldwide. In Denmark, the occurrence is estimated to be 2% [25]. No macrolide resistance was identified in our childhood cohort.

We found radiological changes in 94% of the chest x-rays taken in this study. The radiological findings were quite diverse, but notably, over 80% of children older than two years had a lobar infiltration while the younger children had significantly more subtle findings. This was in accordance with an Italian prospective childhood study [9].

Even in older children, symptoms could not be distinguished from CAP caused by other pathogens. Radiological findings in *M*. *pneumoniae* pneumonia were not distinguishable from CAP in general.

Almost 25% of all children had some kind of rash (erythema/hives) during the illness, and 33% had gastrointestinal symptoms like nausea and or vomiting. Severe extra-pulmonary manifestations accompanying respiratory infections caused by *M*. *pneumoniae* are expected to occur [26]. Two children in our cohort were diagnosed with SJS, which is a known complication of *M*. *pneumoniae* [27]. Outbreaks of *M*. *pneumoniae*–associated SJS in children has recently been reported [28]. We did not see any children with neurological symptoms in this cohort which would be expected.

## Conclusion

*M*. *pneumoniae* is a significant cause of LRTI in children and can cause infections difficult to distinguish from CAP caused by other respiratory pathogens. *M*. *pneumoniae* also affect infants and young children in epidemic periods, and we believe that *M*. *pneumoniae* must be considered as a differential diagnosis to respiratory virus infections and as a cause of infant and childhood troublesome lung symptoms and pneumonia. Chest x-rays were without pathognomonic features to CAP. During an up-coming epidemic, assessment of extra-pulmonary manifestations, especially immunological based skin reactions, can be helpful when diagnosing *M*. *pneumoniae* infections.

## Supporting information

S1 Dataset Anonymized version of database. (XLSX)
